# Supplementary material for: Are carbon emissions trading and green financial instruments synergistic? -Comprehensive quantitative research based on content analysis
Source: PLoS One. 2024 Mar 7;19(3):e0298601. doi: 10.1371/journal.pone.0298601 (PMC10919601; doi:10.1371/journal.pone.0298601)
Supplement: S1 Data — (ZIP) [file pone.0298601.s001.zip › Data/地级市Z_F工作报告.zip!/╜¡╬≈/╞╝╧τ╩╨/└┤╫╘╣1⁄2╓┌║┼í╛┬φ┐╦╩2╛▌═°í┐_2014.docx]

各位代表：

　　现在，我代表市人民政府向大会作政府工作报告，请予审议，并请各位政协委员和其他列席人员提出意见和建议。

　　一、回顾2013年：攻坚克难，经济社会发展稳中有进

　　2013年是贯彻落实党的十八大精神的开局之年，也是我市经济社会发展较为艰难的一年。一年来，面对资源枯竭和产能过剩的双重压力，市人民政府在省委、省政府和市委的正确领导下，在市人大、市政协的监督支持下，坚持城市转型主战略，锐意进取，扎实工作，经济社会保持平稳较快发展。预计全年完成生产总值792亿元，增长8.2%；全社会固定资产投资830亿元，增长20%；规模以上工业增加值360亿元，增长9%；财政总收入109.79亿元，增长9.2%；社会消费品零售总额234.2亿元，增长11.7%；城镇居民人均可支配收入23489元，增长10.5%；农民人均纯收入11100元，增长11%。

　　一年来，主要做了以下工作：

　　(一)致力推进“工业强市”，工业发展更加稳健。深入实施工业强市和质量兴市战略，全力推进“双十”工程，加速推进新型工业化，工业经济稳步增长。

　　工业经济效益和活力不断增强。煤炭、陶瓷、水泥、花炮、冶金五大传统产业全年预计实现增加值299.16亿元，占全市规模以上工业增加值的83.1%。新材料、新能源、生物医药、先进装备制造等战略性新兴产业完成增加值81.36亿元，占全市规模以上工业增加值的22.6%，增长9.15%。全市229户规模以上高新技术企业完成增加值138.4亿元，增长11.7%，占全市规模以上工业增加值的38.4%。全市新增规模以上工业企业70户，规模以上工业企业完成主营业务收入1450亿元，增长8.5%，实现利税总额240亿元，增长8.3%。全市民营企业9722户，新增1169户，民营经济占经济总量的67.5%。

　　园区产业逐步壮大。深入推进园区产业化、集群化发展。全市6个工业园区22个产业基地已有企业503家，完成主营业务收入883亿元，增长21%；完成工业增加值240亿元，增长25%；上交税金38亿元，增长22.5%。全市工业园区(产业基地)主营业务收入占全市工业总产值的61.1%。

　　科技创新能力日益提升。全面推进科技创新“六个一”工程，科技综合实力和创新能力跨入全省先进行列，成功获批全国创新型城市试点市，连续8年获全国科技进步先进市，5个县区全部通过全国科技进步先进考核和专家评审。与中南大学签订了共建萍乡粉末冶金科技园协议。江西省工业陶瓷标准技术委员会落户芦溪县，填补了我市无标准化技术委员会的空白。人才培育和引进力度加大，“院士经济、博士产业”不断发展。江西省工业陶瓷工程技术研究中心博士后工作站获得国家批准，成功创建“全国电瓷产业知名品牌示范区”。萍乡经济技术开发区科技创新公共服务平台被批准为“国家级中小企业公共服务示范平台”。成功获批国家级科技园区(基地)3个、省级园区(基地)6个，专利申请量560项，授权量380项。在第三批“赣鄱英才555工程”评选中，有18人和1个科技创新团队入选，连续三年列全省各设区市之首。有1人入选国家“千人计划”创新人才长期项目。

　　招商引资和重点项目建设成效明显。坚持“四个对接”和“东融西接”招大引强，全年共引进省外5000万元以上项目155个，实际进资274亿元，增长15%。与20余家央企良性对接，共引进央企11户，落户项目17个，总投资额246.5亿元，其中中节能与我市在节能环保领域展开全方位合作，总投资80亿元；深圳比克电池与安源客车合作投资打造5亿元的汽车电池项目；成功引进粉末冶金项目25个，有4个项目投产，12个项目进入孵化园孵化。全市实际利用外资2.45亿美元，增长15.37%。外贸进出口总额12.8亿美元，增长15.17%，其中，出口总额12.55亿美元，增长13.47%。全年列入省重点工程项目31项，新增24项，新增数位列全省第二。实施市重点项目96个，总投资974.3亿元，当年完成投资188.2亿元。中泰纺织项目一期建设完成，国微陶瓷项目一期将于今年元月竣工，华能安源电厂“上大压小”新建工程进入设备安装阶段，熊猫烟花项目竣工，莲花150万吨旋窑水泥项目建成投产，华美立家项目扎实推进。萍洪高速完成13公里路基垫层，昌栗高速动工建设，萍莲高速项目正开展前期工作。杭南长高铁萍乡段完成铺轨，正在加紧电力和信号系统建设。国、省道升级改造里程居全省前列。城区天然气置换工程全面竣工。

　　(二)致力统筹城乡发展，新型城镇化进程加快。全市新型城镇化建设项目达459个，开工390个，完工227个，累计完成投资260亿元。

　　城乡规划日臻完善。基本建立起以市域发展战略规划、城市总体规划、分区规划、控制性详细规划、专项规划、村镇规划为主要内容的城乡规划体系。完成了“一轴两核多组团”发展战略规划、中心城区交通快速通道项目规划等编制工作。全市所有乡镇均编制了总体规划，为确保城镇化建设品质打下了良好基础。

　　城镇化组团大力推进。玉湖新区、田中新区、安源组团基础设施建设全面展开，完成建设投资近60亿元。玉湖新区道路网络基本形成。城投大厦正在装修，安源大剧院、能源中心即将竣工并投入使用，聚龙公园、市民公园、萍实公园、吴楚大道二期等正在建设；玉湖新区城市综合体、城市大厦、玉湖公园、城市综合馆等正在进行前期准备工作。田中新区已完成大部分征地拆迁工作，安置小区已建成7.4万平方米；完成田中湖开挖近200万立方米，景观工程即将开工；杭南长高铁萍乡北站枢纽工程有序推进，站房主体工程完工，站前高架即将完工，洪山大道、站东路路基基本拉通，吴楚北大道、尚贤路、大兴路、站西路大部分路基基本成型，正在进行垫层施工。安源组团中环西路、中环南路完成投资7.11亿元，迎宾大道延伸工程即将收尾，萍安大道南延、萍水河三期治理等工程快速推进。麻山组团启动了省道S234、萍麻路拓宽改造工程；芦溪西部生态组团启动了路行至武功山大道改造等工程；上栗南部组团华洋物流已进入前期施工，碧桂园项目扎实推进；莲花莲江新区组团启动了莲江两岸建设。

　　城市创建扎实推进。积极推进智慧城市建设“十个一”工程。努力争创全国文明城市，启动了城市交通疏堵工程、公园景观形象提升工程、萍水河净化美化亮化工程，公共服务设施不断完善，市容市貌明显改善，城市管理水平明显提升。2013年全国城市文明程度指数测评中，我市城市公共文明指数在地级提名资格城市中排名第八，未成年人思想道德建设工作排名第二十四，两项成绩位列全省地级市第一。

　　生态环境不断改善。全年完成减排项目63个，化学需氧量、氨氮排放量同比均下降1%，二氧化硫、氮氧化物在消化当年新增量的基础上，排放量与上年持平。完成造林绿化12.27万亩，创建“森林乡镇”10个、“森林村庄”100个，提升通道绿化127公里，建成绿道154.1公里，新建(新增)花卉苗木基地2.3万亩，发展省级林业产业化龙头企业14家，造林面积核实率居全省第一名，荣获全省“森林城乡、绿色通道”建设工作先进设区市。市县两级均获全省森林防火平安先进单位。3个乡镇荣获国家级生态乡镇。全年空气质量继续保持在国家规定的二级标准，出境水保持在国家规定的Ⅲ类水质。耕地保护连续3年实现占补平衡。用地用矿秩序好转，实现了卫片执法检查“零问责”。地质灾害防治成效明显。

　　(三)致力夯实农业基础，农业实现稳产增收。在遭受五十年不遇的严重旱灾情况下，坚持大季受损小季补，最大限度弥补农业损失，促进农业增效、农民增收。

　　农业经济稳定增长。预计全年全市农业总产值85.7亿元，农业增加值54.7亿元。完成粮食种植面积125.12万亩，总产57.6万吨，实现了总产“十连增”，单产在全省“十连冠”。粮食收购量达到近10年来最高，为农民增收达1200余万元。现代农业建设稳步推进。农业产业化龙头企业143家，其中51家省级以上龙头企业全年实现销售收入67亿元。农民专业合作社1315家，增长32.3%，家庭农场建设30家。

　　农田水利设施建设力度加大。山口岩水利枢纽工程大坝主体工程全面完工，枢纽电站正式并网发电。整合资金1.6亿元落实高标准农田建设面积3.66万亩。小(二)型病险水库除险加固工程新开工26座；小农水2012年计划项目全面竣工，改善灌溉面积12.56万亩，新启动5479万元投资计划；农村饮水安全工程已解决10.11万农村人口安全饮水问题；中小河流治理项目等城乡防洪工程顺利推进。

　　富裕和谐秀美乡村建设效果初显。投入资金9000余万元加快富裕和谐秀美乡村示范带建设，农村危房改造任务4550户全部完成。可视范围内荒山造林2100亩，水稻、蔬菜瓜果、养殖、油菜、莲子、水产等一批集中连片基地发展到300余个。307个新农村建设点扎实推进，全部完成村庄规划，完成改路242.6公里，改水5.83万户，改厕1.22万户，改房18.82万平方米，使用清洁能源2899户。全市农村公路里程达到6200公里。农村清洁工程以及农村扶贫开发工作有序推进。

　　(四)致力发展现代服务业，第三产业稳步发展。

　　旅游业取得新突破。按照“一头两翼、首尾呼应”的发展格局，全面加快武功山、安源、荷花博览园等景区创A建设。在全省率先建立赣西区域旅游合作联盟。成功举办了第27届全国荷花展暨国际荷花学术研讨会、第六届武功山国际帐篷节等节庆活动，莲花县、安源区顺利入选全省旅游综合改革试点县区，环鄱阳湖国际自行车赛首次进入武功山。成功获评“中国最佳休闲旅游城市”。全年接待游客1706.78万人次，增长21.8%，实现旅游综合收入102.8亿元，增长28.79%。旅游工作“六大工程”建设有序推进，旅游公路大环线项目已列为省交通重点项目。完成了芦万武旅游公路、武功山一级索道更新及中庵区块整治、杨岐游客服务中心、孽龙洞洞内改造等项目。萍乡旅游集散中心已经建成，安源文化博览园、红领巾少儿活动基地、万龙山西海温泉度假村、中国莲花文化园等文化旅游休闲项目稳步推进。

　　文化商贸物流业持续发展。预计全年全市文化产业主营业务收入达180亿元，完成增加值58亿元，文化产业增加值占GDP比重达7.25%，位列全省第一。凯天动漫申报为国家重点动漫企业，安源境界、平步青云等动漫公司落户动漫产业园区，各县区至少建设一个三厅以上数字影院工作正式启动。湘东彩印包装基地申报为省印刷包装基地。编制了《萍乡市现代物流发展规划》，大力发展大型商品交易市场和商贸综合体。联合中国物流与采购联合会共同举办了萍乡市现代物流产业招商对接会。推进了特殊商品口岸作业区建设和公共保税仓建设。赣湘国际物流港一期工程年前全面竣工。金盾物流成为我省首家公共保税仓库。新春蕾粮食物流中心、湘东工业陶瓷、芦溪电瓷口岸物流等平台建设扎实推进。

　　金融业平稳发展。全市人民币存款余额621.71亿元，增长14.9%；人民币贷款余额378.76亿元，增长20.83%。信贷结构更加合理，中长期贷款不断提高，信贷业务逆势上扬。小微企业信用体系试验区建设取得实质性突破，被人民银行总行列为首批国家级试验区挂牌对象。新引进村镇银行1家，新增小额贷款公司1家。政银企合作取得实效，签约金额达193.73亿元，增长35.24%，履约率100%。保险行业覆盖面不断拓宽，保费规模列全省四小市之首。

　　(五)致力民生社会建设，人民群众幸福指数逐步提高。

　　积极保障和改善民生。民生工程全面落实，完成了省政府下达的民生工程指标。就业创业工作成效显著。以成功创建“全国创业先进城市”为契机，形成了市、县区、乡镇(街)、社区(行政村或管理处)四级劳动保障工作平台。全市新增城镇就业3.1万人，新增农村劳动力转移就业2.3万人，城镇就业率达96%，登记失业率3.15%，创业带动就业比率达到1:4.4，零就业家庭就业安置率100%。社会保障体系不断完善。以基本养老、基本医疗、最低生活保障制度为重点，不断扩大社会保障覆盖面，养老、医保、低保等社会保障水平连续十三年位居全省前列。全市城镇基本养老保险、城镇职工基本医疗保险、城镇居民基本医疗保险、失业保险、工伤保险参保人数分别完成年任务的100%、106.9%、108.4%、101.3%和105.3%。社会救助水平进一步提高，城市低保和农村低保保障标准分别提高至每人每月420元和220元。城镇“三无”人员供养标准每人每月提高100元，农村五保对象每人每年提高500元，重点优抚对象抚恤标准平均提高15%。全市城镇基本医疗保险参保率达99%；城乡居民养老保险参保率为90%；新农合参合率为98.6%。全市廉租住房、新建公共租赁住房、廉租住房补贴全部按省政府时间节点开工建设或发放。全市保障性住房3406套全部开工建设；廉租住房租赁补贴完成12581户；棚户区改造完成9.49万平方米、1355户，中央下放煤炭棚户区改造项目已建成9950户，在建5108户。

　　各项社会事业协调发展。萍乡高专成功升本，并正式更名为“萍乡学院”。高考再次取得新突破，素质教育均衡发展。芦溪县荣获全国“两基”工作先进县。江西工业工程职业技术学院成功搬迁。规划投资5亿元、占地400亩的新城区教育园区工程正式启动。文化艺术工作取得新突破，采茶戏《有事找老杨》在第五届江西艺术节首场演出，电影《黄海怀》完成后期制作。市图书馆、莲花县图书馆获评国家一级馆，“新安源·新形象”广场群众文化活动获第十届中国艺术节项目类“群星奖”。安源傩舞、上栗皮影戏等6个项目列入第四批省级非物质文化遗产名录。全市农家书屋出版物补充更新76家。医药卫生体制改革稳步推进，国家基本药物制度全面实施；卫生服务能力进一步提升，服务体系更加完善，市妇幼保健院新院竣工并投入使用，市医院三期建设工程进展顺利；医疗保障和服务水平明显增强，基本公共卫生服务均等化、重大疾病预防控制和免费救治工作成效斐然，湘东区艾滋病防治和社区公共卫生服务工作获得全国先进；中医药事业健康发展。成功创建了食品药品安全城，公共卫生和食品药品形成了安全平稳态势。制定了《萍乡市公共体育设施专项规划(2013-2020)》，全民健身公共体育设施进一步完善，全市社区公共体育设施覆盖率达到73%，农村达到54%。市体育中心改造工程基本完成，成功举办第十二届市运会。低生育水平继续保持稳定，人口出生率控制在13.3‰以内。积极盘活国有资产，妥善处理国企改革遗留问题，国有资产管理体制进一步完善。档案事业快速发展。民族宗教工作规范化、法制化程度不断提高。妇女儿童发展主要目标综合考评列全省第一。住房公积金归集工作提前两年半完成“十二五”规划目标。人防工程扎实推进，国防动员和后备力量建设取得新进步。

　　社会保持安全稳定。全面实施安全生产“企业可防、政府可控”工程，24家煤矿完成了二级标准化矿井建设，12家煤矿采用正规方式采煤；1000条鞭炮企业机械化生产线投入使用，安全湿法制引得到推广，基本实现鞭炮产业安全化、机械化生产。全市工矿商贸领域事故死亡人数占省安全生产控制指标的33.4%，煤矿继续实现“零事故、零死亡”，严厉打击非法生产，安全生产态势持续平稳。推进领导干部接访、下访制度化常态化，按照“项目化管理”要求和“五查”专项交叉督查行动化解信访积案，取得了“五个为零”的好成绩。社会治安明显好转，社会公众安全感、治安好转率、群众对公安工作满意度均名列全省前茅。深入开展了“六五”普法和“法治萍乡”建设。

　　(六)致力提升行政效能，政府自身建设得到加强。

　　民主法治建设不断加强。自觉接受市人大法律监督和市政协民主监督，进一步完善市政府领导领办人大代表建议、政协提案和报告工作、听取意见制度。办理人大代表议案、建议87件，政协提案128件，办复率100%。

　　干部作风更加务实。严格执行中央和上级关于改进工作作风、密切联系群众的各项规定，严格落实党风廉政建设责任制。深入开展“学先进、转作风、强服务、促发展”教育实践活动，引导全市广大党员干部深入基层“入户结亲”，办实事好事3565件。全市“三公”经费支出持续下降，公务支出管理更加规范。全面公开了市本级部门预算和“三公”经费预算。集中开展了“四风”问题和收受“红包”问题专项整治活动，积极组织了对重点领域、重点部门、重点项目、重点资金的审计监督，加强了对党政领导干部的经济责任审计。加强政务公开和政务服务的监督检查，深化风险岗位廉能管理，加大科技防腐力度，建设并运行了电子政务、网上审批、公共资源交易、财政资金收付、廉能风险电子监控、农村“三资”监管、视频监察等七大电子监察平台，政风行风和干部作风明显改善。

　　发展环境进一步优化。进一步提高审批效率，对国务院、省政府取消和调整的行政审批事项进行了衔接清理，强化了行政审批集中办理和联审联批工作，行政审批时间再缩短20%。深入开展优化发展环境集中整治，查处并曝光了一批损害经济发展环境的不良行为。全面开展挂牌服务企业活动，积极为企业解决生产经营中的实际困难和问题。扎实推进绩效管理，不断提升政府效能，规范权力运行。

　　各位代表，面对严峻的挑战、艰巨的任务，我们不等不靠，开拓奋进，实现了经济发展稳中有进、城乡发展日新月异、社会民生建设不断提速的目标。这是省委、省政府和市委正确领导的结果，是市人大、市政协、萍乡军分区全力支持的结果，是各级、各部门齐心协力、共克时艰的结果，是全市人民和社会各界团结一心、扎实工作的结果。在此，我代表市人民政府，向全市广大工人、农民、知识分子和干部及历任老领导、老同志，向各类企业和纳税人，向各民主党派、工商联、无党派人士和社会团体，向驻萍人民解放军、武警官兵、公安干警，向所有关心、支持、参与萍乡建设和发展的同志们、朋友们和海内外友好人士，表示衷心的感谢和崇高的敬意!

　　各位代表，在肯定成绩的同时，我们也清醒地看到，我市经济社会发展中还存在不少困难和问题。主要表现在：经济总量偏小，大项目进度不快，产业核心竞争力有待进一步增强；经济发展质量还有待提升，产业结构需进一步优化，淘汰落后产能亟待推进，经济转型升级任务仍然艰巨；资金、土地、技术和人才等依然是制约经济社会发展的瓶颈；社会民生建设水平与全面建成小康社会的目标仍有较大差距；安全生产、社会稳定等发展中的深层次矛盾和问题仍然严峻，加强和创新社会治理任务十分繁重；一些体制机制有待进一步改革和完善；政务环境有待进一步优化，部分政府工作人员的素质、能力、作风与人民群众期望还有不小的差距；等等。这些困难和问题亟待我们在转型发展中努力解决。

　　二、奋战2014年：改革创新，积极有为促进转型升级

　　2014年是全面深化改革的关键之年，也是实施“十二五”规划的攻坚之年，全市要全面贯彻落实党的十八大、十八届三中全会精神和省委十三届七次、八次全会精神，继续坚持稳中求进的总基调，围绕“一二三四五”的总体工作思路和“一轴两核多组团”的重大战略举措，解放思想，实事求是，积极有为，努力打造改革开放先行先试区、创新驱动转型升级区、民营经济领先发展区和高铁经济引领三化互动区，抢占发展升级制高点。

　　今年全市经济社会发展的主要预期目标是：生产总值增长10%，财政总收入增长13%，全社会固定资产投资增长18%，社会消费品零售总额增长13.5%，外贸出口增长8%，利用外资增长10%，城乡居民人均可支配收入分别增长10%和11%，居民消费价格总水平涨幅控制3.5%左右，万元生产总值能耗下降3%，人口自然增长率控制在9.05‰以内，城镇登记失业率控制在3.5%以内。

　　今年重点做好九个方面的工作。

　　(一)突出改革创新，激发发展动力和活力。认真贯彻落实十八届三中全会精神，正确处理好政府和市场的关系，推进简政放权和重点领域改革，建立健全各项体制机制，正确、有序、协调推进改革，争当改革开放先行先试区。

　　深化市场体系改革。深化工商登记制度改革，实施“先照后证”和注册资本认缴制度，实现工商注册制度便利化。鼓励、支持、引导非公有制经济发展，保证各种所有制经济依法平等使用生产要素、公平参与市场竞争、同等受到法律保护。建立统一开放、竞争有序的市场体系，着力清除市场壁垒，提高资源配置效率和公平性。

　　深化行政体制改革。按照中央和省委的要求，三季度完成市县两级政府机构改革。加快行政审批制度改革，推进审批程序“流程再造”，加大力度取消和下放一批行政审批事项，建立行政审批事项目录管理制度。打破部门利益藩篱，完善加快县域经济发展的政策措施，配合省启动省直管县体制改革试点。加快事业单位分类改革，抓紧出台政府向社会购买公共服务的实施办法。完善国有资产管理体制，以管资本为主加强国有资产监管，改革国有资本授权经营体制，建立健全县区国资监管机构，构建大国资监管格局。

　　深化财税金融体制改革。加快建立现代财政制度。健全财力与事权匹配的市以下财政体制，适当增强市本级宏观调控能力。探索民间资本依法发起设立中小型银行等金融机构，发挥信贷投放主渠道作用，规范发展表外融资，发展多层次资本市场，推进企业境内外上市和中小企业股份转让系统挂牌直接融资。推动改革性保险扩面，营造良好金融生态环境。

　　加快土地利用管理改革。深化土地利用管理制度改革，进一步规范征地程序，完善征地补偿办法，落实被征地农民的社会保障。逐步建立城乡统一的建设用地市场。加快农村集体建设用地和宅基地确权、登记、发证工作，推进农村土地流转制度改革。建立农村产权流转交易市场，鼓励支持承包土地向专业大户、家庭农场、农民合作社流转。完善城市建设用地收储制度，从严打击违法用地和违法建设现象，确保依法严管土地。

　　创新体制机制推进示范区建设。以转变职能、理顺关系、优化机制为重点，加快示范区建设。突破区划壁垒，创新区域合作体制机制，促进与长株潭经济区协同发展，打造赣湘开放合作试验区。全面推进科技入园入企，大力打造创新驱动转型升级区。加强改革和完善民营经济发展体制机制，打造民营经济领先发展区。以高铁通车的经济效应为着力点，打造高铁经济引领的三化互动区。

　　(二)突出产业转型升级，提升工业发展质量和效益。坚持传统产业与战略性新兴产业“双轮驱动”，加快创新驱动，推进集约发展。力争实现规模以上工业增加值405亿元，增长12%；主营业务收入1600亿元，增长13%；利税总额265亿元，增长12%。

　　推进产业转型升级。做好国家资源枯竭型城市转型绩效考核工作以获取下一轮国家转型政策支持。依托新技术、新工艺和新设备，着力改造提升“黑白灰红金”五大传统产业，落实落后产能退出机制，化解产能过剩，加快传统产业改造升级。加快形成以五大传统产业为龙头，以粉末冶金、先进装备制造、环保陶瓷成套设备制造等新兴产业为支柱，以新材料、新能源、生物医药和现代生产性服务业为支撑的现代工业体系，培育一批超10亿元、50亿元和100亿元以上产业集群。

　　加大园区建设促进产业聚集发展。围绕320国道百里经济带“一轴”和“一区五园二十个产业基地”建设，加强园区产业定位和产业链规划，加强园区聚集整合和配套设施建设，引导生产要素向园区基地集中、资源向园区基地整合、资金向园区基地流动，提高产业现代化水平。重点抓好1-2个综合实力好、带动力强的工业园区，培育一批成长性好、经济带动力强的工业园区，打造产业发展新高地。

　　深入推进科技创新。推进产学研合作，重点在主要产业园区(基地)创建科技园区，加快建立市、县区、园区、企业四级科技服务平台。力争全年实施科技重点项目8项以上，开发新产品和高新技术产品100项，认定高新技术企业10家，打造5家创新企业和创新团队，引进10个以上院士、博士项目落户，引进技术成果20项以上，建设2个科技园区，打造2个专业化创新平台。推进我市省级环保产业示范基地建设，力争获批国家级环保产业基地，将萍乡中南大学粉末冶金科技园建设成为国家级科技“孵化器”。

　　(三)突出招商引资和项目建设，增强发展后劲。加快招商引资和项目建设步伐，实施重大项目带动战略，增强经济发展的后劲和活力。

　　抓好招商引资。落实《招商引资三年计划》及相关配套政策。继续推进“四个对接”、“东融西接”推进产业招商、重点招商，围绕“一轴两核”，依托产业园区，突出抓好城市基础设施、商贸市场、现代物流、金融服务、现代农业等项目招商，瞄准国内外知名企业，引进一批好项目，带动和完善上下游产业链条。力争全年引进5000万元以上项目超过169个，增长10%，其中亿元以上项目72个，增长11%。

　　加强开放平台建设。发挥比较优势，积极融入赣西经济转型加快发展战略、赣南等原中央苏区振兴战略。加大对本地生产型外贸企业的支持力度，积极引导我市优势企业外出参展和投资考察。确保商检办事处上半年正式开检，申报设立海关取得实质性进展。

　　加快推进重大项目建设。认真做好国家对萍乡市循环经济试点工作的考核验收工作。争取将萍乡列入第五批国家“城市矿产”示范基地，将莲花县列入中央国家机关对口支援特殊县范围，积极对接萍乡城区老工业区整体搬迁改造项目和生活垃圾无害化处理项目。重点抓好华能安源电厂、航空工业集团(萍乡)汽车零部件、安源比克电动汽车、冠能光电、圆融光电等一批重大工业项目；加速推进大仓前、青草冲输变电等电网重点项目的建设投产。积极推进萍乡陶瓷产业基地铁路专用线、华能安源电厂铁路专用线等重大项目建设。

　　(四)突出“一轴两核多组团”，打造城市建设升级版。按照“一年出亮点，两年出轮廓，三年见成效”总体要求，努力将“一轴两核多组团”打造成为城市新亮点，不断提升城市品位。

　　科学完善城乡规划。全面完善城乡规划体系，编制完成以“一轴两核多组团”为主要内容的都市区总体规划。全面落实“规划一张图、审批一支笔、监管一把尺”，加强重点区域、重要节点的城市设计，保障好“两核”31个重大建设项目的规划服务，确保城镇化建设的品质、品位。全面推进建设规划、基础设施和公共服务向农村延伸，促进城乡一体化，加强农民建房的引导和管理，提高村镇规划建设水平。

　　统筹推进“一轴两核多组团”建设。加快推进中环路建设，确保吴楚大道、毕升路、益民路二期等道路一年内通车。突出抓好“两核”，玉湖新区抓好城市大厦、城市综合馆等公建项目建设，确保安源大剧院正常运营，行政中心完成搬迁，萍实公园、市民公园与行政中心同步完工，翠湖花园、教育园区等项目三年内建成。田中新区建设商周古城遗址公园、聚龙公园、奥体中心、吴楚大道、洪山大道及湖区周边高档综合服务配套等项目。加快推进安源组团、老城区组团、麻山组团、芦溪西部生态组团、上栗南部组团、莲花莲江新区组团建设。

　　构建大交通格局。推进高速公路网建设，实现萍洪高速年底竣工通车，加快推进昌栗高速建设，启动萍莲高速前期工作。加快杨岐至武功山、武功山至明月山两条旅游公路建设，加速推动国、省道改造升级。全面实施农村路网建设，确保“十二五”末实现农村公路“组组”通目标。加强杭南长高铁枢纽工程调度，确保今年10月前竣工通车。积极争取萍浏铁路、萍乡南北向铁路等重大交通项目。

　　(五)突出发展现代农业，促进农村经济增收增效。坚持以发展现代农业为重点，不断改善农业基础设施，构建新型农业经营体系，建设富裕和谐秀美乡村，促进农业增效、农民增收、农村发展。

　　改善农业基础设施。实施好农业综合开发项目，完成4万亩高标准农田建设，抓好10万亩中低产田改造。完成26座、新开工20座以上病险水库除险加固工程，完成万龙山乡、东桥镇等6个乡镇防洪建设及莲花县城下游段防洪工程，新开工六市乡、金山镇等6个乡镇防洪工程。发挥山口岩水利枢纽工程的防洪、供水、发电、灌溉功能。推动农机装备更新升级。抓好标准化设施大棚、规模化畜禽养殖场、健康养殖池塘、标准化蔬菜生产园等建设。

　　提升现代农业发展水平。确保粮食种植面积稳定在120万亩、总产量56万吨以上，加快现代粮食流通产业发展，加强粮食储备体系建设，保障粮食安全有效供给。建设50个以上高标准、高起点、规模大、市场竞争力强的绿色农业生产基地。集中建设1个市级现代农业精深加工产业园区。启动1个国家级现代农业示范园创建，巩固提升4个省级现代农业示范园建设水平，全面铺开市县级现代农业示范园区建设。打造一批农业示范园、农业观光园、农业主题公园、观光采摘园和休闲农庄等各具特色的休闲农业精品，实现休闲农业从业农民年均收入增长30%以上。

　　构建新型农业经营体系。培育一批新型职业农民和专业大户，发展一批家庭农场、合作农场、农民合作社、合作联社，壮大一批农业产业化龙头企业。力争专业大户增长15%，达到1850户；家庭农场翻两番，达到120家；农民合作社增长20%，达到1570家；市级以上龙头企业达到150家，年销售收入突破70亿元。发展多种形式规模经营。

　　抓好富裕和谐秀美乡村示范带建设。加大产业扶持和资金筹措力度，最大限度整合农业政策资金，全面铺开5条示范带建设。把镇村联动作为示范带建设的龙头工程，以镇带村，以村促镇，实现镇村联动发展。在抓好示范带覆盖区域内的32个集镇特别是示范带干线上19个集镇的镇村联动的同时，全面加强“十强中心镇”建设。着力推进农村扶贫开发工作，重点抓好特困片区莲花县和省定123个贫困村及市定集体经济收入薄弱村的扶贫攻坚工作。

　　(六)突出打造消费型城市，增强现代服务业的拉动力。推进以旅游商贸文化等现代服务业为重点的消费型城市建设，力争服务业增加值占GDP比重提高1个百分点。

　　加快发展旅游休闲业。继续实施全市旅游工作“六大工程”建设，加快推进旅游综合改革试点县改革和创建旅游强县，大力推进旅游强市建设。加强武功山和安源景区创5A和杨岐山景区申报国家级风景区、孽龙洞景区创4A工作，完成荷花博览园创4A工作，进一步理顺景区体制机制。武功山景区今年完成5A景区申报工作，争取2015年创建成功。万龙山温泉年内正式运营。安源景区加快推进游客中心、红领巾少儿活动基地、国家矿山公园等项目建设步伐。尽快启动杨岐山普通寺扩建项目一期工程。推进连接核心景区的旅游公路大环线项目建设。继续完善市旅游集散中心服务功能。加快发展乡村旅游，培育一批省级高星级乡村旅游示范点，开展十大乡村旅游示范点评选。打造好武功山国际帐篷节、武功山自行车赛、中国莲花·莲文化旅游节等有影响力的节庆品牌。

　　大力发展商贸物流和文化产业。建设层次清楚的贸易中心体系、特点鲜明的贸易街体系、品类完善的商品交易市场体系、网络健全的现代物流体系和业态先进的零售网络体系。重点推进翡翠城、美佳华等城市综合体的建设，提升商贸服务业档次。加快推进安源中华供销城建设。积极推进物流标准化建设，推进传统商业物资储运企业向现代商贸物流配送中心和专业化、规模化方向发展，引进具备整合供应链能力的嵌入式第三方物流，推动发展农产品农社对接、农超对接等产销模式，推进华洋物流园区、强泰物流等物流项目建设。加快布局文化产业园区建设，推进中国安源文化博览园建设。组织湘东彩印包装产业基地申报国家级印刷包装产业基地。加快安源文化创意聚集区、武功山文化产业园建设。

　　积极发展金融业。加大金融支持实体经济发展力度，进一步加强信用体系建设，力争成为首批国家级信用体系建设试验区，在全市推广小微企业信用体系建设工作。鼓励企业进入新三板，力争在直接融资上有新突破。引进银行业金融机构在萍乡设立分支机构，确保萍乡农村商业银行年内正式挂牌。发挥保险业在资产配置方面的作用，提高保险业在公共服务等方面的效率，积极推进政银企三方良性互动，筹划银团贷款模式，力争今年集中授信额度超过500亿元。

　　(七)突出生态文明建设，建设宜居宜业美丽萍乡。加强生态建设和环境保护，深入推进文明城市和智慧城市创建，发展绿色循环经济，推进节能减排，让百姓的生活环境更加美好，努力建设宜居宜业美丽萍乡。

　　改善生态环境。落实最严格耕地保护制度，确保全市耕地总量动态平衡。推进造林绿化逐步由山下绿化向山上造林转变，突出抓好10个山上造林示范点建设，继续实施全市性封山育林，抓好森林资源管理和森林防火工作，推进主要交通干线可视范围荒山荒坡的绿化，继续实施“森林城乡、绿色通道”建设，推进森林县区、乡镇、村庄创建，不断改善生态环境质量。加强矿产资源管理，严厉打击违法用地和非法开采。加强地质灾害防治，实施绿色矿山建设和矿山地质环境恢复治理。完成县、区污水处理厂二期和工业园区污水处理厂建设；完善污水收集管网，提高现有生活污水处理厂处理效率；推进集镇生活污水处理设施建设，确保每个县区完成1个以上集镇的生活污水处理设施；加强全市饮用水水源地保护，确保饮用水源水质达标率100%。

　　推进节能减排。继续通过工程减排、结构减排、管理减排等措施，全面完成主要污染物减排任务，为全市经济发展提供环境容量。构建生态文明气象综合保障体系，推进大气污染联防联控工作，提高空气质量。完成工业污染源减排、农业源减排、机动车减排任务。确保完成万元工业增加值能耗下降5%；化学需氧量、二氧化硫、氨氮和氮氧化物排放量分别下降2.2%、4%、1.5%和0.5%。争取全国节能减排综合试点城市试点。

　　全力抓好城市创建。全民动员，不断加大创建全国文明城市力度。不断提升群众文明素质，强化城市公共文明指数测评，做好迎接全国文明城市创建总评工作。完善公共基础设施建设。抓好城市绿化，全面启动国家级森林城市创建工作。巩固省级卫生城市成果，策划好新一轮国家卫生城市创建工作。抓好城市公共信息平台、城市公共基础数据库、智慧城管、智慧水务和智慧政务等一批智慧城市项目建设。推行城市管理综合执法。进一步推进城市交通治堵工程，改善城市交通状况。

　　(八)突出发展民生社会事业，加快小康社会建设步伐。以人民幸福为第一追求，统筹推进民生社会事业，加大民生投入力度，集中力量办好一批为民办实事项目，不断提高保障和改善民生水平。

　　全力落实民生工程。坚持把保障和改善民生作为构建和谐社会的重要抓手，深入实施《萍乡市保障和改善民生实施纲要(2011—2015年)》，全面完成省政府下达的民生工程任务和市政府的民生建设任务。实施就业优先战略和更加积极的就业政策，促进创业带动就业，做好以高校毕业生为重点的青年就业工作和农村转移劳动力、城镇困难人员、退役军人就业工作。全市新增城镇就业3.2万人，新增农村劳动力转移就业1.67万人以上，消除“零就业家庭”。逐步缩小城乡、区域、群体之间的待遇差距。基本建成制度完善、管理科学、覆盖城乡的社会保障体系，稳步提高社会保障能力和水平。大力推进“五险”统一征缴，不断扩大社会保险覆盖面，积极落实新型农村合作医疗和城镇居民基本医疗保险政策，扎实推进新型农村社会养老保险。提高社会救助水平，大力发展社会养老事业，加快建立社会养老服务体系和发展老年服务产业，不断提高农村“五保”对象生活水平，稳步提高城乡低保、城镇“三无”、精简退职人员等困难群众保障标准。推进残疾人社会保障体系和服务体系建设，做好残疾人康复、抚养中心建设。按照省下达的“十二五”期间住房保障规划要求，完成保障性安居工程2800套，棚户区改造9123户，加强保障性住房配套设施建设和物业管理。

　　全面发展各项社会事业。深化教育领域综合改革，努力办好人民满意的教育。全面推进学前教育、特殊教育、民办教育、继续教育改革发展。加快新城区教育园区建设步伐，调整新老城区教育网点布局，使中小学校、幼儿园建设与新型城镇化同步协调。加强教育管理和教师队伍建设。培育和践行社会主义核心价值观，提升文化软实力。深化文化体制改革，繁荣文化事业。组织创作一批群众喜闻乐见的文化节目。逐步完善公共文化服务体系建设，推进公共文化服务体系示范区申报工作。做好全国第一次可移动文物普查工作。继续深化医药卫生体制改革，进一步加强卫生服务能力建设，完成市医院三期工程建设并投入使用，抓好市精神病院建设，提高医疗保障和医疗服务水平，大力推进基本公共卫生服务均等化和大病保险制度，为人民群众提供更加安全、有效、方便、价廉的医疗卫生服务。继续稳定低生育水平，稳步推进计划生育“单独”二孩政策。推进食品药品监管体制改革，提升食品药品安全监管能力，确保饮食用药安全。深入贯彻《全民健身条例》，切实加快大型体育场馆建设进程。全面落实优抚政策，争创新一轮全省双拥模范城和全国双拥模范城。加快档案文化建设。加强工青妇、老龄、民族宗教、国防动员和民兵预备役工作。

　　创新社会治理。全面落实安全生产“党政同责、一岗双责”，积极实施“专业治安”和“科技兴安”工作方针，继续推行安全管理“三大工程”和“企业可防、政府可控”工程，煤矿强力推行“站栏式”管理和正规方法采煤；花炮重点推行“6+3”管理措施；非煤矿山行业严格实行“一台两化”管理机制；危险化学品行业强力推行“五符合”管理；其他行业领域落实“1+1”社会综合安全管理，着力加强燃气安全监管，建立纵向到底、横向到边的监控体系，不断提高企业本质安全水平，着力提升政府安全监管能力。健全重大决策社会稳定风险评估机制。健全公共安全体系，完善防灾减灾救灾体制。建立“四访四化”信访工作新机制，构建“统一领导、部门协调，统筹兼顾、标本兼治，各负其责、齐抓共管”的信访工作格局。提升公众安全感和群众满意度，深化平安萍乡建设。加强普法教育和特殊人群管理，加大预防青少年违法犯罪力度，促进法治萍乡建设。强化网络虚拟社会管理，健全网络舆情监管体系，做好突发事件和社会热点敏感问题的网络舆论引导。

　　(九)突出转变政府职能，创造优质高效的政务环境。进一步解放思想，加强和改进政府自身建设，努力建设为民、务实、清廉政府。

　　推进依法行政，强化民主法治意识。进一步提高政府行政决策的科学化、民主化、法制化水平，用法治思维和法治方式管理各项事务。规范行政执法、行政复议和行政调解工作，切实保护公民、法人和其他组织的合法权利。主动接受市人大法律监督和市政协民主监督，充分听取各民主党派、工商联、无党派人士及各人民团体的意见，广泛接受社会公众和新闻舆论监督。实行重大问题市民听证、专家咨询、合法论证、风险评估和社会公示制度，不断提高工作的前瞻性、科学性、实效性。依法推进政务公开和信息公开，发展基层民主，加强社会组织民主机制建设，充分保障人民群众的知情权、参与权、表达权、监督权。

　　优化发展环境，强化服务意识。进一步落实市委、市人民政府《萍乡市进一步优化发展环境的若干规定》的要求，建立规范、科学、有效的优化环境长效机制。建立健全科学、规范的绩效管理指标体系。强化视频监察和网上审批电子监察系统，提高政务服务水平。进一步规范中介机构监督管理。强化服务意识，完善优化发展环境监测工作，重点开展“民调评政”活动，促进行政机关由审批型向服务型转变。

　　加强作风建设，做到勤政廉政。扎实开展群众路线教育实践活动，引导干部“入户结亲”，着力解决“四风”问题。认真贯彻落实中央关于作风建设的规定，认真落实《党政机关厉行节约反对浪费条例》，严格控制和压缩行政成本，实现“三公”经费负增长，严格确保专项资金专款专用。坚持标本兼治、综合治理、惩防并举、注重预防，完善惩治和预防腐败体系。深入开展省市各项重大决策部署落实情况监督检查，加强对公共资金的审计监督。全力实施“权力阳光”工程，推进权力运行程序化、公开化，不断改进工作作风，做到勤政廉政。

　　各位代表!党的十八大和十八届三中全会为我们描绘了新的宏伟蓝图，新的形势赋予了我们新的神圣使命，让我们更加紧密地团结在以习近平同志为总书记的党中央周围，在省委、省政府和市委的正确领导下，全面深化改革，以更加昂扬的斗志，更加务实的作风，团结奋进，攻坚克难，为与全国、全省同步全面建成小康社会而努力奋斗!
